# High shear stress induces atherosclerotic vulnerable plaque formation through angiogenesis

**DOI:** 10.1093/rb/rbw021

**Published:** 2016-06-26

**Authors:** Yi Wang, Juhui Qiu, Shisui Luo, Xiang Xie, Yiming Zheng, Kang Zhang, Zhiyi Ye, Wanqian Liu, Hans Gregersen, Guixue Wang

**Affiliations:** ^1^Key Laboratory for Biorheological Science and Technology of Ministry of Education, State and Local Joint Engineering Laboratory for Vascular Implants, Bioengineering College of Chongqing University, Chongqing, 400030, China; ^2^Taiji Group Co, Ltd, Chongqing, 401147, China

**Keywords:** high shear stress, angiogenesis, outward remodelling, vulnerable plaque, vascular smooth muscle cells

## Abstract

Rupture of atherosclerotic plaques causing thrombosis is the main cause of acute coronary syndrome and ischemic strokes. Inhibition of thrombosis is one of the important tasks developing biomedical materials such as intravascular stents and vascular grafts. Shear stress (SS) influences the formation and development of atherosclerosis. The current review focuses on the vulnerable plaques observed in the high shear stress (HSS) regions, which localizes at the proximal region of the plaque intruding into the lumen. The vascular outward remodelling occurs in the HSS region for vascular compensation and that angiogenesis is a critical factor for HSS which induces atherosclerotic vulnerable plaque formation. These results greatly challenge the established belief that low shear stress is important for expansive remodelling, which provides a new perspective for preventing the transition of stable plaques to high-risk atherosclerotic lesions.

## 1. Introduction

The current review focuses on the vulnerable plaques observed in the HSS regions. Evidence is provided to support that ACS and ischemic strokes occur at or near the proximal region of the stenosis. Arterial diseases such as acute coronary syndrome (ACS) and ischemic strokes are the leading causes of death worldwide [1]. ACS and ischemic strokes are frequently caused by rupture of vulnerable plaque leading to thrombus formation and distal cessation of blood flow. The morpho-mechanical characteristics of vulnerable plaques are critical for their tendency to rupture [2, 3]. ACS and ischemic strokes often occur at sites where the stenosis level is lower than 50% [4, 5]. Atherosclerotic plaque rupture or damage of the vascular surface leads to incomplete or complete obstructive thrombus formation and ultimately cause ACS or ischaemic strokes [6–8]. Vulnerable plaques have the following pathological characteristics: (1) A huge lipoprotein core being larger than 40% of the plaque volume; (2) A thin fibrous cap [9]; (3) High content of inflammatory cells (including macrophages, T lymphocytes and mast cells) [10, 11, 13]; (4) Reduced number of vascular smooth muscle cells (VSMCs); and (5) Plenty of new born blood vessels in the plaques [12, 13].

**Figure 1.**
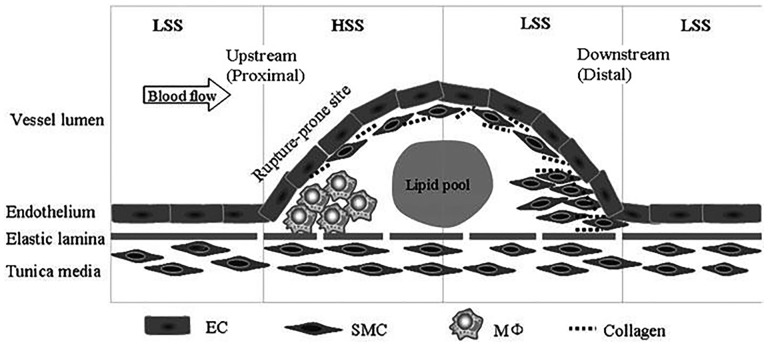
When plaques protrude into the lumen, high shear stress (HSS) is formed at the proximal end of the stenosis also whereas low shear stress (LSS) is formed at the distal part ^[16]^.

Shear stress participates in the formation of atherosclerosis, vascular remodelling, plaque stability, and restenosis after stent implantation and in intimal hyperplasia after blood vessel grafting [14, 15]. The magnitude and spatial distribution of SS change with the development of the plaque [16–18] (Table 1). When plaques protrude into the lumen, high shear stress (HSS) is formed at the proximal end of the stenosis whereas low shear stress (LSS) is formed at the distal part [16, 19 and 20] (Fig. 1).

**Table 1.** The magnitude of HSS and LSS

Term	Location	Magnitude	The relationship with atherosclerosis	Reference
High shear stress (HSS)	The proximal region of plaque	>25dyn/cm^2^	Proathero-sclerotic plaque rupture	[16][17][18][32]
Low shear stress (LSS)	The distal region of plaque	<10-15dyn/cm^2^	Proathero-sclerosis	

## 2. Vulnerable Plaque Animal Model for Shear Stress Research

Change of SS is a critical external factor for the plaque characteristics [21]. Therefore, a proper experimental model of atherosclerotic vulnerable plaques is fundamental for our understanding of SS-mediated vulnerable plaque formation [22]. One model used perivascular carotid collar placement, which rapidly induced atherosclerosis in apolipoprotein E-deficient or low-density lipoprotein receptor-deficient mice [23]. Our group has previously demonstrated the efficacy of this model in studying the development of atherosclerotic plaques induced by SS [24, 25, and 28]. The collar develops HSS in the proximal region and LSS in the distal region of the plaque similar to the plaques intruding into the lumen [26]. Cheng and coworkers improved the perivascular SS modifier that induces regions of lowered, increased and lowered/oscillatory shear stresses in mouse carotid arteries [27, 28].

Another model is ligation of the left external and internal carotid branches. In this situation left carotid blood flow is reduced to flow via the occipital artery. In response to partial ligation of the left carotid artery (LCA), blood flow significantly decreased by 90% in the LCA and increased by 70% in the right carotid artery (RCA) [29–31]. The major advantage of the first model is the similarity to well-defined plaques, the accelerated atherosclerosis formation and induction of at least two kinds of SS stimulations simultaneously. HSS occurs inside the stenosis and low and/or oscillatory SS is localized to the distal region of the stenosis. However, the change of SS in the proximal and the distal regions is more serious with the ligation model which can induce very LSS in the ligated LCA. However, the ligation model is not localized, i.e. the SS is changed throughout the vessel.

## 3. High Shear Stress Induces Vascular Outward Remodelling

Vascular remodelling encompasses chronic changes of the vascular lumen size and morphology, vessel wall structure, and vascular function [32]. SS induced vascular remodelling is a very complex process involving nitric oxide (NO) expression, extracellular matrix (ECM) synthesis and degradation, and VSMCs proliferation and migration.

### 3.1. High shear stress up-regulates the expression of NO

NO is an important vasodilator participant in vascular remodelling [33]. Endothelial cells (ECs) are the main sensor of the SS and also the critical player in vascular remodelling [34]. In the early stages of atherosclerosis, LSS occurs at the two sides of an arterial bifurcation and on the inside of vascular curvatures whereas HSS occurs at the apex of an arterial bifurcation [35] and on the outside of the vascular curvature [36]. In resistance arteries as well as in large blood vessels, chronic increase in blood flow enhances endothelial nitric oxide synthase (eNOS) expression and NO-dependent vasorelaxation [28, 32–39] whereas LSS decreases endothelial NO synthesis [28]. Furthermore, reduction in blood flow induces inward remodelling and reduced arteriolar contractility [33, 40]. Moreover, NO is essential for arterial outward hypertrophic remodelling after a chronic rise in flow [33, 41]. In addition, NO can induce ECM degradation through increasing the expression of matrix metalloproteinases (MMPs) [33]. This remodelling allows the effect of altered SS on the vascular wall to be normalized [42]. Therefore, HSS induces vascular outward remodelling through increasing NO expression [43, 44].

### 3.2. High shear stress induces the degradation of ECM

ECM synthesis and degradation plays an important role in vascular wall remodelling [45]. MMPs regulate vascular remodelling by ECM degradation [46]. Hence, the study of SS regulating the expression of MMPs can clarify the understanding of vascular remodelling under SS [47]. HSS induces MMPs expression and vascular outwards remodelling [48]. The likely mechanism involves NO in MMPs expression where HSS induces NO synthesis [28, 36–39] and NO increases the expression of MMP [49, 50]. HSS also induces secretion of plasmin (a strong specific activator protein for MMP-specific precursors secreted by macrophages) [51]. In addition, Pro-MMP-2, activated MMP-2, and proMMP-9 levels were modestly increased by high flow after 7 days [51]. Therefore, HSS may induce high MMPs expression [52]. MMPs promote plaque wall structural changes, severe internal elastic lamila (IEL) degradation. This provides a channel for inflammatory cell and SMC invasion which in turn produces intensive MMPs to degrade collagen and elastic fibres. These processes lead to severe wall and lumen expansion and may be the cause that the HSS region forms a thin fibrous cap in vulnerable plaque on the proximal side of the vascular stenosis [19, 53].

### 3.3 High shear stress induces the apoptosis of VSMCs

Under physiological conditions, SS does not directly act on VSMCs. However, when medial VSMCs migrate into intima after endothelial injury, they become directly exposed to blood flow [54]. The studies in apoE-deficient mice have revealed that VSMCs in atherosclerotic plaques are derived exclusively from the local vessel wall rather than from circulating progenitor cells [55]. LSS induces VSMCs migration into the intima in the ECs-VSMCs coculture model [56]. Therefore, LSS may be important for VSMCs proliferation and migration and for promoting blood vessel wall thickening which all are factors leading to atherosclerosis stenosis formation [57]. LSS-associated intimal hyperplasia was dependent on platelet endothelial cell adhesion molecule-1 (PECAM-1) [58], suggesting that PECAM-1 is necessary for flow-induced vascular remodelling.

High laminar SS inhibits SMCs proliferation and promotes the apoptosis of VSMCs [59]. This has been demonstrated as a direct factor for vulnerable plaque formation [60]. The finding is consistent with the clinical finding that apoptosis of VSMCs is mainly localized in the HSS region of the stenosis [61–63]. Therefore, vulnerable plaques are mainly found where SS is high because HSS induces apoptosis of VSMCs [48, 51, 64]. In vessel grafts, increasing SS inhibits smooth muscle cell proliferation and reduces intimal hyperplasia [65, 66]. The mechanism could be linked with Bone morphogenetic protein 4 (BMP4) [67] and NO [68] signalling pathways. HSS promotes release of endothelial NO mediating apoptosis of VSMCs [66, 69]. HSS also upregulated the expressions of NF-kappa B phosphorylation and MMP2 and MMP9, facilitating vascular outward remodelling [70]. SS induces vascular NADPH oxidase to comprise p47phox but not gp91phox. Generated Reactive oxygen species (ROS) interact with NO to produce peroxynitrite, which in turn activates MMPs and facilitates vessel remodelling [71]. ECs have an important regulatory role in the biological behaviour of VSMCs [72]. HSS promotes progressive arterial remodelling, which consequently causes blood vessel rupture [35, 73]. In summary, HSS induces adaptive and serious outward vascular remodelling through promoting apoptosis of VSMCs [74].

### 3.4 The remodelling process under shear stress

Systemic factors such as hyperlipidemia, hyperglycemia, and hypertension and genetics [75] exacerbate the local HSS and inflammatory response and may facilitate the transition of early atherosclerotic plaques into high-risk plaques. Vascular remodelling is governed to maintain the previous (normal) SS. For example the brachial artery remodels to maintain local SS despite the presence of cardiovascular risk factors [76].

After plaque formation and protrusion into the lumen, HSS is mainly apparent in the proximal region of the stenosis whereas LSS is at the distal region [19, 77]. HSS leads to the expansive remodelling [78, 79], which is a compensatory process [30, 80]. Expansive remodelling in response to chronic or repetitive flow increase involves a coordinated sequence of events in the arterial wall as extensively reviewed by others [81–83]. HSS induces aneurysmal remodelling through vascular expansive remodelling for maintaining the local SS [35, 36, 84]. Research showed that HSS increased the vascular diameter by 23%, while LSS reduced the diameter 23% [37, 85]. Outward remodelling is the critical factor for high-risk plaque formation [32, 86, 87]. NO release from ECs exposed to excessive shear is a fundamental step in the remodelling process. NO potentially triggers a cascade of events, including growth factor induction and MMP activation that together contribute to remodelling of the vessel wall [88]. Furthermore, high flow rates not only induce HSS but also increase cyclic strains which are found to induce arterial expansive remodelling [89]. Evaluation of vascular local SS and cyclic strain was used to predict vascular remodelling and plaque development [90].

Although several reports show that LSS promotes vascular expansive remodelling [27, 75, 91–93], both clinical and animal models prove that vascular expansive remodelling mainly localizes in the HSS region [29, 94]. The increased atherosclerotic wall thickness in HSS regions is associated with loss of compensatory remodelling [95]. Vascular remodelling maintains luminal SS stability; hence excessive outward expansion is the direct way to reduce the local HSS.

## 4. High Shear Stress Induces the Vulnerable Plaque Formation

In vivo colour mapping with intravascular ultrasound and magnetic resonance imaging (MRI) data show that coronary plaque rupture are localized in the arterial regions with elevated SS [64, 97–102] (Table 2). Animal models confirm that vulnerable plaques mainly occur in the HSS region of the stenosis [62, 103].

**Table 2.** High shear stress induce rapture-prone plaque formation or rapture in clinical report

Sample	Proximal	Shear stress	Phenomenon	Device (detected method)	Reference
Twenty patients	Proximal	High shear stress >25dyn/cm^2^	Increase necrosis area	Virtual histology-IVUS and CFD	100
A 67-year-old woman	Proximal	High shear stress >32dyn/cm^2^	Lipid/necrotic core, intraplaque hemorrhage	MRI at 10-month follow up	97
20 patients	Proximal to the point of maximum stenosis	Blood wall pressure was 82 ± 18 mm Hg	Coronary plaquerupture	3-dimensional IVUS	98
119 patients	Proximal to the point of maximum stenosis	Higher than the distal	Ulceration	Angiographic ulceration	103
42 human carotid atherosclerotic plaques	Proximal to the point of maximum stenosis	Higher than the distal	Apoptosis in the distal	Immunohistochemical (anti-CD31, anti-Ki-67)	110
12 patients	Proximal	38.9 versus 14.4 dyn/cm^2^	Ruptured plaques	MRI	101
12 patients	Proximal	>25dyn/cm^2^		Angiography and IVUS	102

A main difference between stable plaques and high-risk plaque is inflammatory cell accumulation [96, 97, 104, 105]. Inflammatory cell invasion into atherosclerotic plaques is modulated by ECs. The recruitment and infiltration of inflammatory cells into the endothelium are mediated by upregulating adhesion molecules, chemokines and integrins [98, 106, 107]. The viewpoint that LSS induces vulnerable plaque formation is based on the high expression of inflammation-related proteins on ECs [27, 107, 108]. However, LSS induces apoptosis of ECs and endothelial dysfunction [64, 109–112]. Hence, it is inconsistent with the established role of LSS in destabilizing atherosclerotic plaques regarding the expression and activity of MMPs [113]. In addition, LSS induces the VSMCs proliferation, migration and ECMs synthesis [114].

At present, the cross-sectional morphological characteristics of atherosclerotic plaque have been extensively investigated. However, less attention was paid to the axial distribution of plaques in the artery. Clinical pathology research shows that vascular plaque rupture mainly occurs in the proximal region of the stenosis, where macrophages aggregate and thrombosis is found under the endothelium [62, 103]. Connective tissue growth factor is released from platelets exposed to HSS and is differentially expressed in endothelium along atherosclerotic plaques [115]. In vivo MRI 3D FSI studies show that 63.5 dyn/cm^2^ SS induces high-risk plaque formation [116]. Taken together, these studies demonstrate that there is a high correlation between HSS and vulnerable plaque formation in the axial direction.

## 5. Angiogenesis May Be the Main Reason That High Shear Stress Induces Atherosclerotic Vulnerable Plaque Formation

A growing body of evidence shows that HSS prevails in the proximal region of atherosclerotic plaques protruding into the lumen [28, 117]. Significant differences in plaque morphology between the proximal and distal parts of plaques indicate a role in arterial flow in the distribution of different cell types [28, 53, 62, 98, 117, 118]. It was shown that 86% of ruptured plaques are located proximal to the stenosis [118]. The reason that atherosclerotic plaque rupture occurs in this region is currently unknown. Oxidized low-density lipoprotein was proposed, because oxLDL activates/induces subsets of smooth muscle cells and macrophages to gelatinase production [62]. However, it is well known that HSS is endothelium-protective and the endothelium may prevent the low-density lipoprotein (LDL) from entering into the vessel wall [19]. Furthermore, some studies showed that oxidized low-density lipoprotein (ox-LDL) is mainly accumulated in the distal region where SS is low [19, 62, 119].

Neovascularization in the vessel wall promotes the formation of atherosclerosis and vulnerable plaque development. The new vasa vasorum (VV) can transport cellular and soluble components such as red blood cells, inflammatory cells and lipid/lipoproteins into the vessel wall [120–122]. A recent report showed that bFGF and VEGFR-2 overexpression in the adventitia induced development of VV and accelerated plaque progression [122, 123]. Furthermore, most microvessels in atherosclerotic arteries were immature with abnormality of intraplaque microvascular ECs with incomplete endothelial junctions and membrane detachment. This may link the association between the microvascular leakage and intraplaque haemorrhage in advanced human coronary atherosclerosis [124, 125].

**Figure 2.**
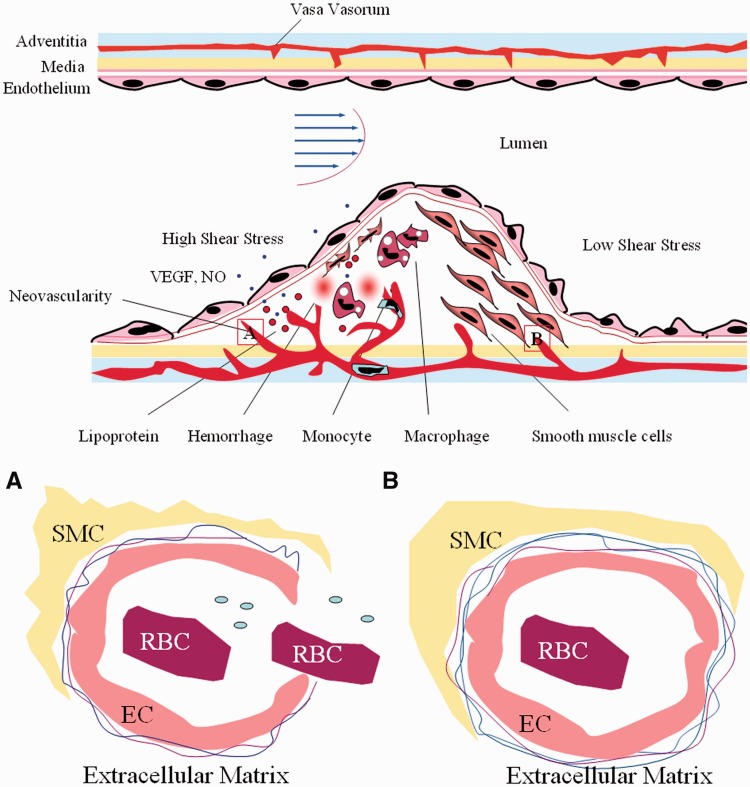
High shear stress induces atherosclerotic vulnerable plaque formation through angiogenesis. High shear stress promotes the expression of vascular endothelial growth factor (VEGF) and endothelial nitric oxide (NO), resulting in angiogenesis of endothelial cells (EC) that form vasa vasorum and increases the endothelial cell permeability. Furthermore, NO induces smooth muscle cell (SMC) apoptosis and matrix degradation, resulting in loss of mural cells and the basement membrane around newborn microvessels. This results in microvascular leakage. The leaky vasculature becomes entry points for inflammatory cells, red blood cells (RBC) and lipid/lipoproteins. This may result in inflammation, intra-plaque haemorrhage, lipid core accumulation and eventually plaque rupture.

HSS plays a critical role in the expression of vascular endothelial growth factor (VEGF) [126] and endothelial NO synthesis [28, 34–36, 68]. VEGF induces angiogenesis [127] and also disrupts the vascular barrier function in diseased tissues [128]. NO mediates shear-induced angiogenesis in ECs [129] and increases vascular permeability [130]. Furthermore, the highest concentration of NO is also critical for the loss of VSMCs and ECM [131]. Thus, HSS causes the ECs to form tube-like structures and increases endothelial permeability by increasing the expression of VEGF and NO. The leaky vasculature with high endothelial permeability and without a restrictive basement membrane exhibits no adequate barrier function (Fig. 2).

We propose that angiogenesis is the reason that vulnerable plaques are localized in HSS regions. Furthermore, NO induced smooth muscle cell apoptosis and matrix degradation. The result is loss of mural cell and basement membrane around newborn microvessels, causing microvascular leakage. The leaky vasculature becomes entry points for inflammatory cells, red blood cells and lipid/lipoproteins. This may result in inflammation, intra-plaque haemorrhage, lipid core accumulation and eventually plaque rupture.

## 6. The Mechanical Mechanism Underlying Plaque Rupture

As pointed out above, SS in the proximal region of stenosis is significantly higher than in the distal region. HSS is critical for vulnerable plaque [132]. Intraplaque haemorrhage is associated with higher SS and higher structural stresses in human atherosclerotic plaques as shown by *in vivo* examining MRI-based 3D fluid-structure interaction [133]. Numerical simulation shows that the SS in the proximal region of stenosis may reach 50-60 dyn/cm^2^ when the stenosis degree is 50%. The SS in the proximal region does not exceed 20 dyn/cm^2^ once 70% stenosis is reached. This may precipitate the rupture of vulnerable plaque in the proximal regions when less than 50% stenosis [130, 134]. Although increased SS in the proximal region may lead to plaque fibrous cap rupture, 75% of the plaque rupture is believed not to be due to SS since the wall SS is much smaller than tensile stress during the cardiac cycle [19].

The haemostatic system is a modulator of atherosclerosis [135]. HSS induces intra-thrombus fibrin deposition and platelet adhesion to the arterial wall [136–138]. HSS also promotes platelet aggregation [139]. Hence the haemostatic dysregulation caused by HSS may contribute to our understanding of why ACS and ischemic strokes are located preferentially in the distal region of the stenosis. SS rate is the rate change of the local SS and it is an important factor for vulnerable plaque rupture [136, 140]. Microfluidics is an important tool for blood clotting [141, 142] where platelets preferentially adhere in low-shear zones downstream of the formed thrombus, with stabilization of aggregates dependent on the dynamic restructuring of membrane tethers [143].

Under HSS conditions, blood pressure decreased and uniaxial tensile stress increased at the site of vascular injury. The magnitude of SS is smaller compared with the overall loading of plaques. Hence, pressure may be the main mechanical trigger for plaque rupture and risk stratification [144]. 3D critical plaque wall stress in prior rupture plaques is 100% higher than that for plaques that do not rupture. However, flow SS is 92.94 dyn/cm^2^ for rupture plaque, which is 76% higher than that for non-rupture plaques (52.70 dyn/cm^2^) [145]. Rupture sites in human atherosclerotic carotid plaques are associated with high structural stresses [146]. Once the thin fibrous cap is formed, the internal stress increased 200% when the fibrous cap thickness decreased by 50% [147]. These results demonstrate that intravascular haemodynamic factors are responsible for the progression of coronary atherosclerosis and development of vulnerable plaques [148]. Autopsy data have shown that there are obvious difference between circumferential plaque stress and vulnerable plaques. The plaque rupture zone is associated with a high degree of stress concentration [149]. Circumferential stress and Young's modulus are important direct factors for plaque rupture [150, 151]. Furthermore, plaque wall stress and flow SS may produce a significant uniaxial strain [152]. Research results have shown that the small pressure difference in the order of 20 mmHg can generate quite a high uniaxial strain in 75 μm thick plaques. Eccentric plaques would be exposed to a more serious uniaxial strain [153]. Hence HSS and the vessel wall thickness are also responsible for plaque rupture [154–156]. In summary, increased wall SS, circumferential stress and pressure are all important for plaque rupture, especially the pressure of the plaque. However, SS is closely related to plaque formation and progression [157].

## 7. Research Perspective

The current review focuses on the vulnerable plaques observed in the HSS regions. Evidence is provided to support that ACS and ischemic strokes occur at or near the proximal region of the stenosis. Having reviewed the published results in the literature, we noted that data on the relationship between SS and plaque rupture is contradictory and inconsistent. Previous research mainly focused on the biological function of SS, and less attention was paid to the mechanical properties of extracellular surroundings and the blood vessel itself [158]. The roles of blood vessels, vessel wall thickness and elastic modulus factors have been somewhat ignored considering plaque rupture [159]. From the literature reviews we can conclude that LSS is the main mechanical factor in plaque formation while HSS may be the main cause for the transition of stable plaques into inflamed lesions. Vascular mechanical stress may be the direct trigger for plaque rupture. How and when do those mechanical stresses function to regulate vulnerable plaque formation and destabilization? And what is the association between blood pressure and mechanical stresses? These issues remain uncertain, but it is quite necessary to further illuminate the molecular mechanisms underlying the plaque formation in response to SS [159–164]. SS and chemical stimuli may synergistically regulate vascular remodelling [165].

Currently, numerical analyses have been effectively used to simulate the physical and geometrical parameters characterizing the haemodynamics of various arteries during physiological and pathological conditions [166–168]. Numerical analysis can contribute to reveal the mechanism for development of plaques and predict the tendency for a plaque to rupture [169, 170]. Moreover, clinical imaging techniques such as magnetic resonance or computed tomography (CT) combined with numerical analysis methods have assisted considerably in gaining a detailed patient-specific picture of blood flow and structure dynamics, which could effectively prevent and treat this disease [171, 172].

## 8. Clinical Implications

SS changes with the degree of stenosis, and the changed stress regulates the development of plaques into high risk plaques [173]. Locally increased SS using a developed flow divider indicates that SS reduces in-stent neointimal formation by 50% [174, 175]. Attempts to increase SS to inhibit intimal hyperplasia are not applicable to atherosclerotic vulnerable plaque treatment [68] because HSS is the critical factor for high-risk coronary plaque formation. After the treatment of stenosis with percutaneous transluminal coronary angioplasty (PTCA) balloon and stent, the SS increases, which promotes vascular outward remodelling. This eventually leads to restenosis or even vulnerable plaque formation [176, 177]. Besides SS, the average wall shear stress (AWSS), average wall shear stress gradient (AWSSG), oscillatory shear index (OSI) and relative residence time (RRT) are important parameters for reducing the number of false positives. AWSS identifies the largest number of plaques, but produces more false positives than OSI and RRT [178]. It is necessary to increase the variety of detection methods, especially to pay attention to the proximal region of the vascular stenosis for detecting the SS [98, 179, 180]. Evaluation of the volume of the plaque is also an indirect method for the SS around the plaque [181]. A 3D fusion of intravascular ultrasound and coronary CT are useful for in-vivo wall SS analysis [182, 183]. It is necessary to combine optical CT tomography and coronary angioplasty *in vivo* for the evaluation of the connection between the SS and the characteristics of vulnerable plaques [180]. Regarding drug development, the regulatory effects of drugs on the SS should be cautiously considered; otherwise it may lead to more serious vascular disease [184, 185].

Lipid-lowering drugs may change the characteristics of plaques and the thickness of blood vessel wall and elastic modulus [186]. The vascular stiffness affects the sensitivity of ECs to SS and thereby participates in the regulation of vascular remodelling [187]. Changes in vascular cyclic stress can also influence SS-mediated vascular remodelling of VSMCs [188]. MRI assessment of plaque biomechanical properties including wall SS and internal plaque strain provides information on early plaque progression and vessel remodelling [189, 190]. More precise magnetic resonance, intravenous ultrasound (IVUS), CT and angiography were applied to analyse and predict plaque development and stability [191]. Morphological and mechanical features should also be considered in an integrated way for more accurate assessment of plaque vulnerability, allowing for early identification of plaques with inflamed phenotypes [191, 192]. Critical plaque stress/strain conditions are affected considerably by stenosis severity, eccentricity, lipid pool size, shape and position, plaque cap thickness, axial stretch, pressure, and fluid-structure interactions. These variables may be used for plaque rupture predictions [193–195].

If our hypothesis that angiogenesis is the main reason that high SS induces atherosclerotic vulnerable plaque formation is true, it may provide new perspectives for clinically predicting the location of plaques vulnerable to rupture and how to prevent plaque instability. Theoretical models could be developed to predict the relationship between the magnitude of SS and atherosclerosis plaque rupture. It also could be applied to arterial bypass grafting through selection of the most appropriate geometry to adjust the SS for reducing the formation of microvessels. Finally, previous studies have shown that plaque microvessels may serve as an interface for plaque expansion. Therefore, we can narrow the range of treatment strategy since plaque angiogenesis is primarily localized in the proximal plaque region.

In summary, SS has been shown to play a role in plaque formation, progression and rupture. The underlying mechanism of plaque formation seems to differ from plaque rupture. Plaque formation is localized in the LSS region whereas plaque rupture occurs primarily in HSS region. HSS induces up-regulation of NO and VEGF of ECs in the proximal region, which leads to microvessel formation in the plaque from VV. Moreover, the pathological angiogenesis is an entry point for infiltration of inflammatory cells, deposition of lipoproteins and the occurrence of intra-plaque haemorrhage. Decreasing the angiogenesis or the leaky vasculature [196, 197] induced by HSS may establish a more favourable microenvironment, which can impede vulnerable plaque formation.
